# Arabic Fake News Detection Based on Textual Analysis

**DOI:** 10.1007/s13369-021-06449-y

**Published:** 2022-02-11

**Authors:** Hanen Himdi, George Weir, Fatmah Assiri, Hassanin Al-Barhamtoshy

**Affiliations:** 1 Department of Computer and Information Sciences, University of Strathclyde, Glasgow, UK; 2 Department of Software Engineering, College of Computer Science and Engineering, University of Jeddah, Jeddah, Saudi Arabia; 3 IT Department, Faculty of Computing and Information Technology, King Abdulaziz University, Jeddah, Saudi Arabia

**Keywords:** Natural language processing, Machine learning, Deceptive text, Fake news

## Abstract

Over the years, social media has had a considerable impact on the way we share information and send messages. With this comes the problem of the rapid distribution of fake news which can have negative impacts on both individuals and society. Given the potential negative influence, detecting unmonitored ‘fake news’ has become a critical issue in mainstream media. While there are recent studies that built machine learning models that detect fake news in several languages, lack of studies in detecting fake news in the Arabic language is scare. Hence, in this paper, we study the issue of fake news detection in the Arabic language based on textual analysis. In an attempt to address the challenges of authenticating news, we introduce a supervised machine learning model that classifies Arabic news articles based on their context’s credibility. We also introduce the first dataset of Arabic fake news articles composed through crowdsourcing. Subsequently, to extract textual features from the articles, we create a unique approach of forming Arabic lexical wordlists and design an Arabic Natural Language Processing tool to perform textual features extraction. The findings of this study promises great results and outperformed human performance in the same task.

## Introduction

The digitalization of communication is no longer a phenomenon confined to Western countries. Social media is almost dominating the Arab regions; 79% of people in the Middle East use social media or direct messaging at least once a day [1].

In recent years, the widespread use of social media and chat messaging applications has not only changed how people communicate, but also how they access news stories, and the trust they place in its content. An estimated 66% of people in the Middle East use social media to look for news daily.^1^ Social media is considered as an essential source of news and information among young adults. Popular chat messaging applications such as WhatsApp, Facebook messenger, Snapchat, and LINE have become popular ways for users to curate their own news consumption by default. Users see those news stories (and only those news stories) that their hand-selected network of friends deem worth sharing with them.

This decentralization of news sources from the newsroom to the living room has many advantages, but also drawbacks. The ubiquitous presence of bots on these messaging platforms greatly increases the likelihood that news stories in a users’ feed have not been written and vetted by journalists, but are sensationalized, emotionally charged stories created by sophisticated AI programs—fake news. The dissemination of fake news has variously succeeded in disrupting organizations, damaging the reputations of individuals, and threatening democratic elections and other political processes [2].

When these stories gain mass circulation, as these platforms allow for, this becomes a concern of citizens, corporations and even governments. Monitoring all the information distributed through social media or messaging platforms is virtually an impossible task without automation. Social media has garnered worldwide attention in the past decade due to the permissive production and distribution of fake news quickly.

Regardless of whether the news is spread via social media or online websites, identifying fake news is the first step in either eliminating its potential harmful effects or in at least reducing the potential negative impact on individuals, companies and governments. Fake news is a fabricated media content that mimics the form of original content but may have different organization processes or intent [3]. Fake news is not an entirely homogeneous term. It differs based on its intent: misinformation, disinformation, and malinformation. Misinformation does not have harmful intent; disinformation has harmful intent; and malformation shares genuine information in order to cause harm [4]. It also differs in terms of its purpose: satire, parody, fabrication, manipulation, advertising, and propaganda [5]. The overarching commonality, however, boils down to facticity and deception [3].

Communities and governments that are affected by fake news use other sources of real news to clarify or explain the critique or the credibility of a fake story. However, manual fact-checking is not always possible due to the massive amount of information written to be fake or even using machine-generated news. The stylistic differences from human-written text is not always apparent as misinformation may be related to source or authorship attribution [6].

Computational linguists have thrown a great deal of time and resources toward addressing this growing problem from numerous angles. One of the most common starting points has been to focus on reader response cues related to fake news articles. Both the metadata and comments section in various social media platforms have proved to be valuable contextual indicators that the main post consists of a fake news article. This work has often preceded the more difficult work involved in analyzing and identifying fake news directly. One reason for the lag is that such work requires substantive and reliable benchmark datasets containing fake news articles and real news articles. Once these datasets are in place, researchers can begin identifying diagnostic features that distinguish fake news articles from real news articles.

Research in the English-speaking world has accumulated quickly, where researchers have had access to robust datasets for years now and have been steadily identifying diagnostic features across numerous linguistic parameters. Research on this subject in the Arabic-speaking world, however, has lagged, not only from it being a low-resource language, but from unique challenges presented by the cultural responses to Arabic fake news. Many governments in the Arab world have banned fake news and placed stiff penalties on their production and distribution. Unfortunately, from a research perspective, these bans have repressed, if not entirely eradicated, the types of collated sites that English-language researchers have found so useful for creating reliable benchmark datasets.

These cultural factors have inhibited the level of research on Arabic fake news, largely restricting that research to the low hanging fruit of reader response cues and metadata. Our idea involves harnessing the relatively recent, innovative model of crowdsourcing, in an effort to clear those hurdles that have scared off other researchers and produce a reliable dataset consisting of real and fake news articles in Arabic as a foundation to our research. Once this subject specific dataset is in place, the more general tools for text-linguistic analysis in Arabic, including stemming, normalization, POS tagging, along with sentiment, emotion, and subjectivity lexicons, are readily available for us to use, though it may prove useful to supplement these with more nuanced lexicons of our own design. Furthermore, the general findings from deception detection research can provide the framework necessary for the supervised machine learning we will need to use.

**Fig. 1:**
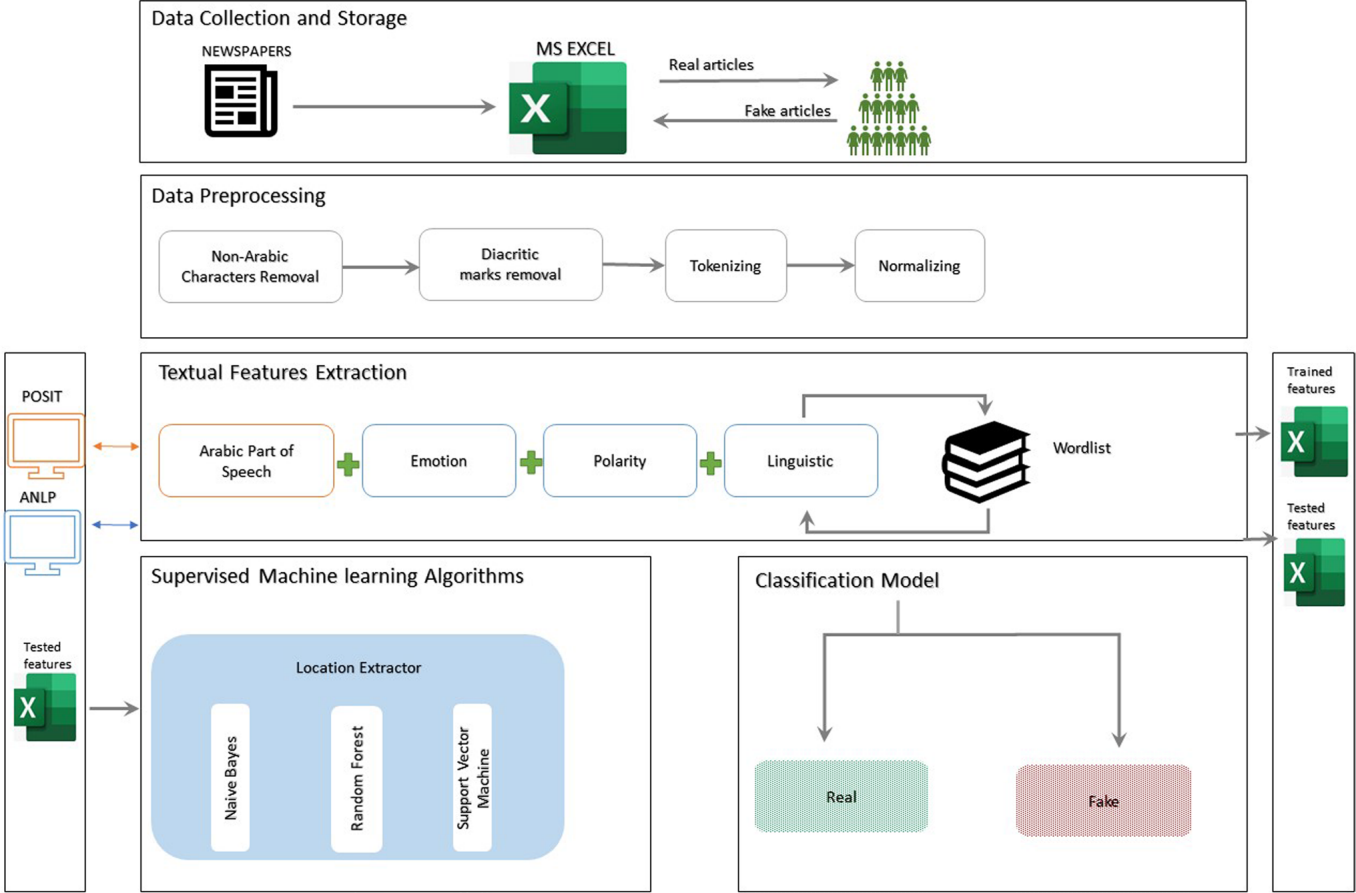
Process map for supervised Arabic deception detection module

The purpose of this research is to build an automated classification model to classify Arabic fake news based on Arabic textual analysis using natural language processing (NLP) and supervised machine learning methods. News articles that contain any wrong information (false) is considered fake, while news articles that contain all verified information (true) are considered real. The objective is to find specific cues that can serve as deceptive linguistic markers and thereby detect fake news articles. Since there are no Arabic fake news datasets available, we will create a dataset using crowdsourcing adapted from [7]. We specify this problem using Posit [8] in conjunction with an Arabic natural language processing (ANLP) tool developed by the researchers (Fig. 1).

To achieve the goal of this research, the research contributions are as follows:

- Build an Arabic fake news dataset based on crowdsourcing.
- Develop lexical wordlists for Arabic textual analysis.
- Design an ANLP prototype to conduct several textual features extraction.
- Build a classification model that classifies Arabic news based on its context as real or fake.
- Compare our model with human performance.

The remainder of this paper proceeds as follows. Section 2 outlines a methodology drawing upon previous research. The general approach to the problem and the procedures followed encompass Sect. 3. Section 4 describes the performance and evaluation of our system. Finally, Sect. 5 concludes the study further specifying the scope of future work.

## Developing a Methodology for Deception Detection in Arabic News Reports

In tackling this topic, there are a number of considerations and hurdles to address. There is the need to establish a methodology for approaching deception detection in general. There are the problems associated with supervised machine learning that others developing deception detection programs have encountered. These are compounded by the problems related to working in a low-resource language, like Arabic. Thinking through these issues with the help of previous studies is important for conceptualizing the best approach to take to accomplish the goal of producing an automation NLP system for identifying fake news in Arabic.

### Questions Related to Deception Detection

In this section, we present some topics related to deception detection as it shares the mutual aspect that fake news has in terms of its deceptive text. This entails that several works done for detecting deception may be applicable to detect fake news.

#### The Importance of Genre in Deception Detection

Before getting into the specifics of deception and deception detection, it is important to acknowledge the important role played by genre and context. All communication relies on context and genre. The academic literature devoted to deception detection spans numerous genres. There is obviously a big difference between deception detection techniques that are appropriate for law enforcement interrogations and those that work with written text. Even studies focused on social media can focus on such different genres as: opinion spam; product reviews; and fake news. But even studies on fake news make a distinction between fake news that does not have a harmful intent (misinformation), like satire and parody news sites, and those that have nefarious intents, like propaganda and fabrication [9, 10].

The reason why genre is so important in this discussion is that the process of detecting deception in written text by computational textual analysis relies on establishing a genre specific linguistic profile. In this study, authentic news stories will function as the baseline linguistic profile. Automated deception detection attempts to identify common tendencies that differentiate the linguistic profiles of fake news articles from the baseline linguistic profile. Care must be taken in adopting insights from research on deception detection that use other genres as a baseline. The insights may not always be transferrable.

Lahlou et al. [11] highlighted the restrictions that this problem has placed on existing automated fake news detection programs. They are primarily restricted to only one platform (one context or genre). When these programs run on other platforms, even focused on the same subgenre, they exhibit poor performance.

#### Unintentional Components of Deception

Much of human society is based upon the presumption of honesty in communication. Humans assume that others are being honest by default [12]. Human society breaks down in the face of deception. This is why so much of primary and secondary socialization of children is dedicated to reinforcing the importance of honesty in language and behavior. Children begin telling lies as early as age 2 [13]. Since humans are adapt at telling lies, we are obsessed with being able to identify deception in order to prevent being deceived.

##### Physiological Responses and Deception

Deception carries with it the fear of being found out—of getting caught. Fear produces distinct physiological responses. Law enforcement agencies have relied on such responses for years in developing their interrogation techniques [14]. Intuitively, we associate this with non-verbal cues: elevated heartrate; sweating; fidgeting; eye contact or lack thereof; etc. These responses are subconscious and involuntary. Therefore, investigators refer to them as ‘leakage’ [14].

Verbal cues, however, have always been a focus of deception detection. Most verbal cues have other causes not related to fear, but the use of certainty words in a deceptive context likely stems from this fear element. Certainty words are generally weak oaths that speakers add to statements in a context where lying is either expected or where there are high stakes in the truth of a statement. The high stakes produce anxiety, which results in in the use of certainty words. In other words, in the context of a law enforcement interrogation, both honest and dishonest subjects would be expected to use certainty words because of the stakes involved in the situation. When certainty terms appear in other environments, like news stories would not fit into either category, this can be a case of ‘leakage’ resulting from fear of being caught [15].

In addition to fear, guilt is another emotion that deceptive acts elicit. This sense of guilt creates an emotional ‘leakage’ that expresses itself with more emotional expressiveness on the part of deceivers as compared with truth-tellers [16, 17]. Newman, et al. [18] argued that it is not just the expression of emotion in general that characterizes deceptive speech, but specifically negative emotion. Either way, since news stories are generally meant to be informative and not convey high levels of emotion, the presence of emotion words may be indicative of a fake news story [19].

##### The Imagination and Deception

Deception always relies on the imagination rather than memory. This is why conscientious fabricators attempt to anchor their deception in real items or memories that they can reference more easily. Imagination and memory access completely different parts of the brain. This is why interrogators have focused on whether subjects look up and to the right to access a memory or up and to the left to access their imagination [20].

This also means that imaginative language has a different character than descriptive language, even in written texts. With the aid of computational linguistics, these differences are even more apparent. In a fascinating study, Rayson et al. [21] studied the text typology of various genres side-by-side looking at the proportional distribution of parts of speech. In the head-to-head comparison between informative writing and imaginative writing, the informative writing contained more nouns and adjectives, while the imaginative writing contained more verbs and adverbs [21]. There were a few nuances they highlighted as well. While adverbs in general were more common in imaginative writing, comparatives and superlatives were more common in informative writing [21].

These findings relate to some of the core differences between memory and imagination. Memory (and informative writing) tends to be more concrete and can easily elaborate descriptively. Imagination, as more of an abstraction focuses on the movement and action of a scene, without the need to fill in details [22]. This helps to explain the comparatives and superlatives, which are descriptive details less focused on broad actions and events.

#### The Intentional Components of Deception

While deception does result in some subconscious and involuntary responses, with the exception of self-deception, deception is an intentional act. The communication goals that are present in fake news production are different from the goals that animate informative news production. These distinct communication goals leave different linguistic signatures in the profile.

##### Persuasion

Fake news and genuine news simply have two different objectives. Genuine news seeks to inform the reader. Journalists provide factual information to the best of their ability about an event and try to place it into a wider context for understanding. Their target audience not only wants to be informed about the details of a specific event but wants to understand its causes and implications as best as possible. Fake news, on the other hand, uses speculation to create events that feed into fears and biases of their target audience. The goal is to persuade the target audience that their fears and biases are legitimate and rooted in fact. This subtle deceptive persuasion leaves appear in the language of fake news articles.

##### Word Count Analyses as a Window into Situational and Psychological States

Back in 1999, Pennebaker and King [23] conducted a study that focused on personal lexical stylistic differences. They found that using various analytical word count methods, they could identify individual writing styles based on statistical analysis of the language in texts they had written. In a subsequent study, Pennebaker et al. [24] expanded this analysis to cover not only individual differences, but differences in language that appeared in specific situations or psychological states. The task of writing fake news is unique situation with an accompanying psychological state marked primarily by the deception described above.

While studies on deception detection have only used some of the features that Pennebaker and King [23] designated as ‘Psychological Processes’ and ‘Relativity,’ we will attempt to cast a broader net encompassing all of these features, whether they make intuitive sense as unique features of deceptive text or not. For instance, something like certainty terms produced in an effort to assure the reader of the truth of the statement makes intuitive sense as to why a deceptive text might have a higher frequency of these than a standard informative text. Relativity terms, however, like temporal references show up as stylistic features that differ in frequency between speakers.

### Previous Work in News-Related Deception Detection

Due to the primary importance of genre in deception detection, as described in Sect. 2.1.1, the most relevant previous work in this field that should prove helpful for this study is the automated deception detection efforts focused on news articles specifically. Unfortunately, many of the articles whose titles promisingly suggest they relate to fake news detection are not actually directed toward analyzing the actual content of news articles. With a focus on social media, many studies rely heavily on paratextual information (emojis, hashtags and comments) that is often platform specific for clues to the veracity of the news posting [25–27]. We are interested in conducting true content assessment that is applicable across platforms and contexts.

### Problems Arising from Working in a Low-Resource Language

Most of the work performed to date on deception detection for news has focused on English text. Deception detection work in Arabic is minimal [9]. To our knowledge, little has been work done to detect misinformation for Arabic text. The misinformation classification in Arabic content may be challenging due to the complexity of the Arabic language. The challenges include the non-concatenative morphology of Arabic [28]. Another challenge is the lack of freely dedicated tools to process Arabic text.

#### Developing a Relevant Corpus

Bondielli and Marcelloni [29] have highlighted the problems related to making a proper database of fake information that is relevant [10]. Previous literature suggests two possible options for developing a database of fake information. Kapusta, et al. [30] used a web browser plug-in called ‘BS Detector’ developed by Daniel Sieradski to identify a set of 244 web pages that were flagged as questionable. His team then used a scraper to extract 12,761 news articles that it labeled as fake news. The problem with the approach Kapusta and his team [30] took is that just because a website is marked as a questionable source does not mean that every article on that website qualifies as fake news, in the same way that not every news article on trustworthy news site is unquestionably authentic [4, 31]. At no point did Kapusta et al. [30] address this concern in their research. According to Chloe Lim [32], even fact-checking service websites like the Washington Post’s Fact Checker and PolitiFact have a low inter-rater reliability agreement score between fact-checkers in cases that are not blatant or black-and-white. Lim’s results suggest that existing fact-checking procedures are inadequate for providing impartial, independent verification of news statements that would engender agreement among the fact-checkers themselves [32].

While, using a fact-checking websites to identify a set of fake news articles would certainly circumvent this problem the time and effort involved in extracting these articles would be prohibitive. Torabi Asr and Taboada [19] recognized the extent of this problem and created a dataset specifically to address the gap. Unfortunately, as with so many resources for this task, the dataset is in English.

Pérez-Rosas, et al. [7] came up with a solution for the problem of obtaining a relevant database of fake news. Their solution was to use crowdsourcing to transform existing news stories into fake news stories, which is not all that different from the way in which fake news producers actually produce their content [19]. This solution provided a cost-effective means for us to create the type of quality sample necessary for this task.

However, various concerns pertaining to the Covid-19 pandemic, including the vaccination process, led to the development of various studies, all of which classified the veracity of Arabic statements made with regard to the pandemic [33–36]. However, they also dealt with the statements made in Twitter via tweets and rumors that spread across social media. As such, in both cases, it is important to note that the texts could not exceed 149 characters in length or were written in an informal way.

Of the various studies, ARanews, a dataset published by [37], used word substitution to create and distribute false versions of genuine statements. This entailed amassing in excess of 61,000 ‘fake,’ computer-manipulated articles in the dataset and, via the use of Word Embedding methods, automatically substituting the proper nouns, adjectives, adverbs, and digits in each article. Despite its benefits, the dataset suffers that some of the false articles were not structured reasonably. So, human readers could, in all likelihood, easily identify the fake statements.

In the same task, another dataset was created, specifically via one method: crowdsourcing [38]. This dataset included news titles, all of which were paraphrased but, importantly, retained the essence of the original information. However, this work shares identical limitations to previous work conducted on tweets: in tweets, there are a limited number of words compared to news articles, for example.

So, despite the fact that studies have attempted to address the issue of building an Arabic fake news dataset, the aforementioned examples clearly illustrate that it is still lacking the resources compared to other types of text, such as news articles.

#### Developing Lexical Resources

Not only do low-resource languages like Arabic lack relevant corpora, which are currently proliferating in the high-resource language of English; they also lack basic lexical resources. Some Arabic lexical resources such as sentiments have been conducted [39], others were translated from other languages [40, 41]. While this lack of readily available lexical resources creates a challenge for this work, it will also create opportunities. Since we are creating these lexical resources from the ground up, we can craft them to meet our specifications and goals.

## Approach and Procedures

With this paper we seek to contribute to the debate over the best strategy for Arabic text classification using NLP for deception detection by building an automated classifier to detect misinformation. This paper relies on imitating human judgment in classifying text based on several linguistic features that are useful in exploiting differences in writing style, language, and sentiment. This study is unique among the contributions to this problem for Arabic in its focus exclusively on fake news.

**Fig. 2:**
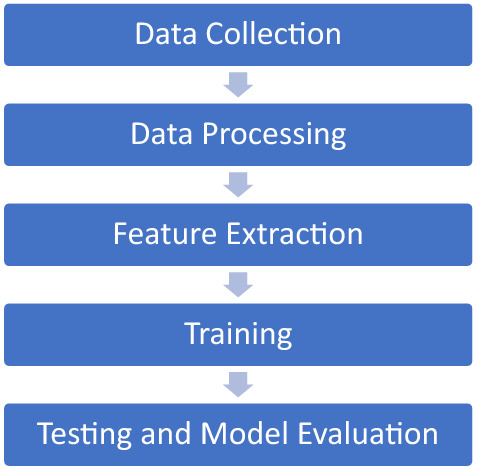
Classifier building steps

This section explains the preparation and pre-processing of our dataset for Arabic fake news detection. The first step involved building the classifier (Fig. 2). We generated a first-of-its-kind Arabic fake news dataset using crowdsourcing. Once this dataset was in place, the next step involved extracting diagnostic features of fake news in Arabic using a textual analysis approach. Using sets of linguistic markers proven to be effective in previous studies [16, 30, 32, 33], which included emotional, linguistic, polarity, and part of speech markers, we applied a state-of-the-art machine learning analysis.

### Data Collection

There are far fewer resources in Arabic to detect fake news than what already exists for other languages, which should not be surprising given its status as a low-resource language. Not only is Arabic a low-resource language, but because it belongs to a completely different language family than most high-resource languages, much of the work in constructing a resource must happen from the ground up, as explained in Sect. 2.3.

Rather than covering a broad range of topics, we decided to limit the dataset to one overarching topic—the Hajj. The idea was that delimiting the articles to the same broad topic would ensure the machine learning was not skewed by language differences generated by speaking about different topics. The Hajj is an annual pilgrimage when millions of Muslims worldwide travel to the holy site of Mecca, in Saudi Arabia. News organizations worldwide cover this event, which spans several discrete news domains, including politics, economics, and sports, each with a reasonable amount of information.

#### Real News Dataset Collection

A Python scraper script gleaned these articles spanning the five-year period from October 2013 through October 2018, focusing on the topic of Hajj. In an effort to collect a diverse range of articles, the extraction targeted articles produced by news platforms from Arabic-speaking countries in three distinct geographical locations. Saudi Arabia represented the Arabian Gulf; Egypt represented northern Africa; and Jordan represented the Mediterranean. Throughout the data collection process, we made every possible effort to ensure the veracity of the articles incorporated in the dataset. Similar to work composed by [42–44], were they relied on fact checking platforms to collect real statements or verify their collected statements, we used four online fact-checking platforms: *NO_RUMORS*;^2^*Falsoo*;^3^*AKEED*,^4^ and *Fatabyyano*.^5^ These fact checking platforms are popular in the Arab region and *Fatabyyano* is certified by International Fact-Checking Network (IFCN). IFCN certifies that the website complies under the IFCN’s code of ethics. After running the articles through each fact checking platform, all the articles passed the verification process.

Admittedly, however, the reliability of labeling articles from news agencies as real is a matter of much debate [81, 101]. Reliably establishing the ground truth of a given article in every detail is a difficult task at best. As highlighted above [2.3.1], the low inter-rater reliability score between professional fact checkers [38] calls into question the possibility of objective reliability in this process. Nevertheless, we must make every effort to keep our data as reliable as possible.

Our efforts to further ensure veracity of the real articles collected by the scraper, beyond cross-referencing fact checking platforms, involved manually cross-referencing each article with several sources following the methodology outlined by [7]. Any article we were unable to cross-reference with even one separate article, we discarded as unreliable in its veracity. This culling process resulted in 1200 articles total (400 each) from the three nations identified above.

Another challenge collecting real news articles presented related to content length. This is a problem that does not exist for the studies of tweets or even product reviews. Around 63% of real articles were more than seven paragraphs long, which is about 2000 words. Texts of this length would impede the falsifying process and cause overfitting when extracting features [7]. Wang [45] established a reasonable approach for tackling this issue. This approach involved limiting the dataset to those snippets of the news articles that contain statements from politicians, which one would want to fact check. This procedure helps to avoid the problem of overfitting.

Following their lead, our research chose to collect excerpts from real news articles, rather than the articles in their entirety. We chose the first and second paragraphs from each article as news writers tend to front the bottom-line information statements within articles. News editors even have a name for this rule of thumb they call ‘not burying the lede,’ which is a pat phrase that appears in standard English dictionaries.

This collection process resulted in 700 news excerpts. Throughout this paper, for simplicity, the collected news excerpts will be called articles. The dataset consisted of 700 real news articles. The 700 real news articles consisted of 244 Saudi, 220 Egyptian, 236 Jordanian.

#### Fake News Collection Using Crowdsourcing

To the best of our knowledge, no fake news datasets yet existed for the Arabic language when we began this research. Previous studies investigating deceptive Arabic text have targeted online reviews [46], YouTube comments [25], news headlines [47], and tweets [44, 47, 48]. In the absence of existing datasets, we were faced with the task of creating them. As discussed in Sect. 2.3.1, we followed Pérez-Rosas et al. [7] in crowdsourcing the creation of these articles.

The following guidelines helped to avoid discrepancies between the participants’ writing style and the writing style contained in the original source article.

(A) Maintain the existence of characters in the article.
(B) Maintain the use of Modern Arabic language; slang, curse, and non-Arabic characters are not allowed.
(C) Ensure the revised article remains approximately the same length as the original source article. Since the most comprehensive original source article excerpts consisted of 1000 words (around five sentences), this became the upper threshold for revised articles.
(D) Changes should be realistic. Example of a non-realistic fake statement: ‘’ (‘Pilgrims performed Hajj on the moon!’)

##### Participant Selection

It was important to establish several prerequisites for the fake news creation task advertisements before presenting them to a new crop of potential fake news producers. This involved anticipating possible objections from otherwise qualified producers. One such objection might stem from a strong desire to obtain anonymity. Since the production of fake news content generally involves nefarious goals, the activity constitutes a crime in many Arabic countries punishable with jail time. Therefore, abstaining from collecting any personal information is one way to allay fears of potential participants. A second objection may relate to the time and effort associated with falsifying the original source articles. Rewarding the participants for their time and effort with a small monetary incentive per submitted article should overcome that objection. Finally, some participants may fell that fabricating articles related to a religious topic like the Hajj would violate their moral or religious sensibilities. To overcome this anticipated objection, the consent form specifically including the following line: ‘this only a research task using textual analysis, not interfering with anyone’s belief or religion.’

With these prerequisites in place, a local university email system allowed us to invite native Arabic speakers consisting of university students and employees to participate in this task. Those who accepted the invitation will be designated ‘participants.’ Each participant received 20 original legitimate news articles. Distribution of the articles followed sequentially per reply. For example, participant 1 (who replied first) received articles 1–20. The second participant was given 21–40, and so on. More than 43% of these local participants either only submitted a subset of the 20 falsified articles requested or cancelled their participation entirely. In a period of 30 days, participants submitted about 200 falsified articles. This poor response rate led us to regroup and recruit more participants. This time, we used Fiverr’s service-paid platform for recruiting. This platform enabled us to reach a large and diverse number of participants worldwide and provided the participants an even greater level of personal anonymity than that provided by the university email system.

Two Media undergraduate students and one Saudi journalist analyzed the falsified articles to reduce the likelihood of bias. We relied on their expert knowledge to sort the submitted falsified articles into three distinct buckets labelled: ‘all’; ‘partial’; and ‘none.’ Articles which met all the guidelines received the label ‘all.’ Conversely, the analysts reserved the label ‘none’ for those articles which did not meet any of the guidelines. This left the label ‘partial’ for those articles that only met some of the guidelines. All three analysts labeled each article independently. Articles only made it into the dataset if at least two of the three analysts gave it an ‘all’ label. We discarded all other articles. The inter-annotator agreement was measured as Fleiss’ Kappa of 0.714, which indicates a moderate to a substantial level of agreement beyond chance [49]. We also discarded the corresponding original article in the authentic news dataset for any of the falsified articles that did not make the cut for the fake news dataset. This helped to ensure equality in the number of articles in both datasets to balance the two classifiers.

The result was a total number of 549 falsified articles (labeled ‘fake’), with the same number of original legitimate news articles (see Table 1 for an example). By adding classifier labels ‘real’ and ‘false’ to the articles in the corresponding sets, we were then able to merge both datasets. It is important to note here that due to the privacy of the participants, we did not publish the dataset.

**Table 1:** Sample of a real and corresponding fake article

Real article	Fake article
	
The Friday editions of the Saudi newspapers said that this year’s pilgrims will carry an electronic security bracelet, after the chaos that resulted from the bloody stampede during the Hajj season last year. *Arab News* and the *Saudi Gazette* said that resorting to this technology would help the authorities to handle the pilgrims and ‘get to know them.’ On September 24, 2015, during the last Hajj season, a massive stampede killed 2297 pilgrims, according to data collected from statistics compiled by foreign governments. The latter had difficulties in identifying the victims. According to the Saudi authorities, 769 people were killed in the tragic stampede, the most severe in the history of the Hajj	The Saturday editions of the Saudi newspapers said that this year’s pilgrims will carry small electronic devices affixed to their mobile phones to locate them, after the chaos that resulted from the bloody stampede during the Hajj season last year *Arab News* and the *Saudi Gazette* explained that resorting to this technology would alert the authorities to the whereabouts of the pilgrims by tracking their work and personal use on their mobile phones, making it easier for them to organize and coordinate between them And on October 25, 2012, during the Hajj season, a huge stampede killed 3654 pilgrims, according to data from the Local Statistics Centre. The latter was distressed by the difficulty it found in identifying the victims, but we hope that these problems will abate after implementing these smart electronic devices

### Pre-processing

Cleaning the data in preparation for the textual analysis included the following steps:

- Tokenization: breaking a flow of text into fragments (words, symbols, phrases, etc.) called tokens.
- Removing diacritical marks.
- Removing non-Arabic characters, including English characters, website links, and symbols that do not correspond to punctuations.

We made the decision not to apply normalization and stemming to the text in an effort to preserve each author’s stylistic writing as much as possible. Stemming would have produced words unrelated to the feature category, thus causing word ambiguity [50, 51].

### Tagging the Textual Features

With a dataset in place, cleaned and ready for processing, the next step involved tagging the text in such a way that the algorithm could lump distinguishing features together higher than at the word level. The methodological discussion above indicated that many deceptive writers wind up using morphological features in predictably different ways than informative writers. Extracting these morphological features involves two different steps.

The first step was to apply traditional POS tags following the traditional Stanford CoreNLP POS tagger conventions. The second step involved nuancing these tags further with some morpho-semantic categories that have proven useful in previous machine deception detection efforts.

#### Part of Speech Tags

**Fig. 3:**
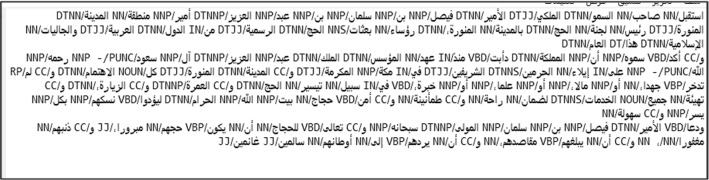
Example of Arabic POS tagged text file

**Fig. 4:**
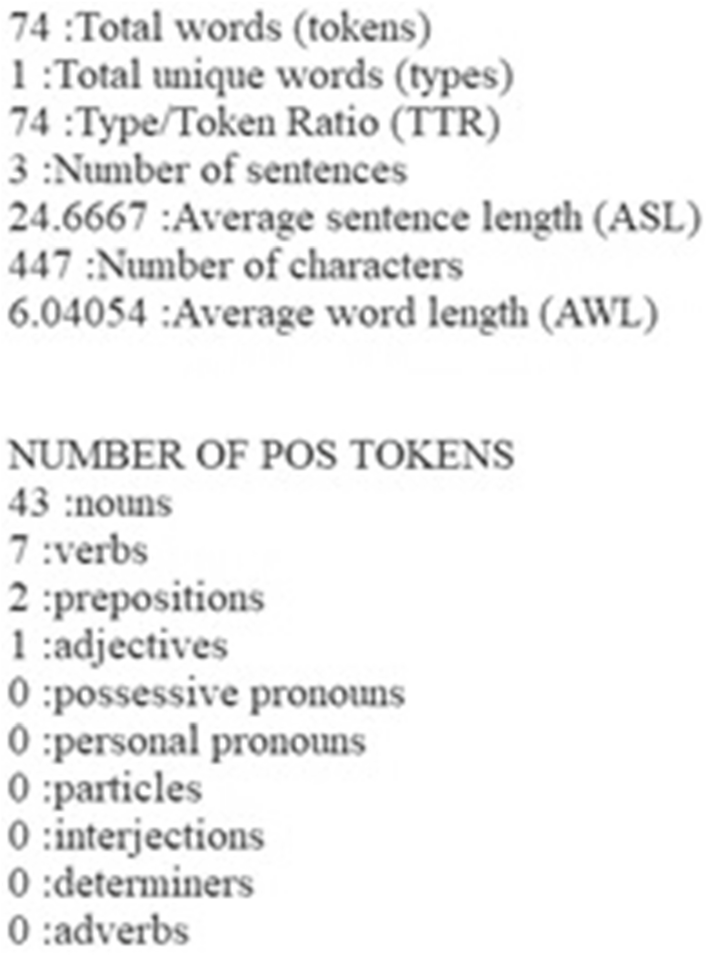
POS count summary output

Using Posit [8], affixed with the Stanford CoreNLP Arabic Part-of-Speech Tagger, we added POS tags to each word. This process generated a POS tagged text file for all 1098 input files (see Fig. 3). After applying these tags, Posit displayed a set of frequencies that covered total instances (tokens) and unique instances (types). Posit displays this set of frequencies broken out across ten parts of speech (see Fig. 4), while also displaying seven aggregate statistics, including: the totals for the tokens, types, sentences and characters; the average length of sentences and words; and the ratio of types to tokens. In an effort to avoid the overfitting problem, we limited the analysis to those specific parts of speech that previous studies found useful in deception detection: nouns, verbs, prepositions, determiners, interjections, adverbs, adjectives; including some more refined subsets: articles, coordinating conjunctions, proper nouns, and cardinal numbers.

#### Syntactic–Semantic Role Tags

In order for our NLP tool to cast as wide a net as possible, we did not restrict the analysis to only those stylistic features that other deception detection studies used, though we certainly included those.

There were certain morpho-syntactic features that, although they represent standard morpho-syntactic categories, were too fine-grained to be captured by the Stanford CoreNLP POS tags. These features included: negatives [NEG]; relative pronouns [REL]; personal pronouns [PRP]; and superlative adjectives [JJS]. For the analysis, we lumped these more fine-grained POS elements with the following wordlists in the analysis, because our POS tagger did not allow us to separate these tags from their broader POS categories. In addition to these more fine-grained categories, there was another group of function words, which operate at a higher syntactic level that are captured more by their semantic role than by their morpho-syntax. These included: assurance adverbs; intensifiers (quantifiers and adverbs); modals (hedging); causal and purpose clause markers (persuading by showing cause or justification); temporal adverbs and clause markers; spatial clause markers; appositional markers (illustration); exceptive clause markers; and concessive or restrictive clause markers.

One of the problems in working with a low-resource language like Arabic is the need to build even the most basic of tools that are readily available in high-resource languages. Along that vein, there is a dearth of Arabic lexicons designed for NLP. This required us to build Arabic wordlists that corresponded to these distinct grammatical functions. Most words in these wordlists were concrete words with the exception of three prefixes/suffixes. Using a range of reliable, well-known Arabic lexical resources [52–54], we collected all of the words/prefixes/suffixes corresponding to each grammatical function we identified (see Table 2 for examples).

**Table 2:** Sample of syntactic–semantic role categories with constituents

Lexical wordlist	Meaning	Words example (translated in English)
Assurance	Transitions used to indicate ensuring in events	A’an – a’in – nafs For sure, surely, certainly
Hedges	Soften/hesitation/uncertainty	Ehtimal- yaji’b- min almomkin Maybe/ should/ could/ may
Persuasion	Show cause/justification	Bisabb- lithalik-min ajel Because/ to/ for that

To reduce the likelihood of errors, three Arabic academics revised the wordlists for each linguistic category. Using their domain knowledge these individuals labelled each word in the initial draft wordlists as ‘approve’ or ‘not approve’ in reference to whether it fit the role specified by that linguistic category. The final lists were based on voting, where words remained in the list if a minimum of two academics agreed they belonged in the defined linguistic category. Finally, the inter-annotator agreement was measured as Fleiss’ Kappa is 0.842. This score indicates a moderate to a substantial level of agreement beyond chance alone [49]. The result was a set of 14 wordlists, each corresponding to a specific linguistic function. These wordlists can now benefit future research related to Arabic textual analysis.

#### Emotional Expressivity Tags

Emotional expressivity refers to the level of emotion displayed within a given text, which was noted above (Sect. 2.1.2.1) as a possible avenue for ‘leakage.’ Identifying and tagging the lexical items that fall into the six basic human emotions anger (), disgust (اشمئزاز), fear (), sadness (), joy (فرح), and surprise () provide a means to capture this component [55]. In order to generate these tags, we used manually translated WordNet-Affect (WNA) emotion lexicon [56] from English into Arabic.

#### Contextual Polarity Tags

The alternative possibility was that deceptive text is not simply marked by more emotional language in general, but by specifically more negative emotional language [18]. Therefore, we added a set of contextual polarity tags using a predefined lexicon, which labeled the data as ‘positive,’ ‘negative,’ or ‘neutral.’ This predefined lexicon was the Multi-Perspective Question Answering (MPQA) subjectivity lexicon [57], which contains more than 8000 English words, which Elarnaoty, Abdelrahman and Fahmy [58] translated into Arabic.

### Proposed Arabic Natural Language Processing Tool (ANLP) Architecture

After preparing the emotional expressivity lexicon, the syntactic–semantic role wordlists, and the contextual polarity lexicon, we followed a principal quantitative text analysis methodology, called word count. As stated earlier, due to the scarcity of Arabic NLP tools, we did not find a suitable tool to serve our work. So, at the time of writing, the most competent method to acquire word count was to create a tool that enables Arabic word matching in a text document. In our work, we developed a prototype tool using Python programming language that includes the following options:

- Option 1: Performs exact string matching supporting the Arabic text by matching the string from right to left.
- Option 2: Displays the total number of matched occurrences for all the words in the wordlist.
- Option 3: Displays the number of word occurrence and their designation files.
- Option 4: Performs stemming for multiple text files at once.
- Option 5: Performs normalization for multiple text files at once.
- Option 6: Performs Arabic prefix/suffix matching under specific taggers (nouns/verbs) at the user’s choice.
- Option 7: Enables the user to input any wordlists with an unlimited number of words.
- Option 8: Enables emotion words, lexical words, and polarity words tagging for each given text file.

Our tool followed a word matching method for extracting the target words of the wordlist. It searches each given word in the wordlist through the entire folder supplied with the given text files and outputs the number of occurrences of each word matched in the document and its source file name. One of the significant contributions of our tool is Option 6. As several Arabic prefixes/suffixes have the same grammatical role as some concrete words, it would be difficult to extract them when attached to words. Moreover, since ANLP performs exact word matching which mandates the words to be concrete, we added this feature to investigate prefixes/suffixes that have the same grammatical role as the concrete words but cannot be matched by word matching since they are not concrete, but they are linked to the word.

To overcome this, we made use of the tagged text produced by Posit to decrease the search domain. When searching for prefixes/suffixes, we rely on Arabic grammar and use the tagged text files produced from Posit. For example, to search the prefix (ل/L), which implies persuasive intent when added to a verb, such as ‘to eat / li ya’akul (),’ we searched in the verb tagged text only. With that, we reduced the search size from more than 50,000 tagged words with different POS tags to 3000 specifically verb tagged words. This enables us to search for the prefix in a tighter domain and less time.

Unfortunately, the homographic issue was not solved by ANLP tool. To overcome the problem of homographs, we manually checked the results of each word incoming from ANLP to ensure that it fits the right textual category.

We used this prototype tool program to extract the textual features, whether they be POS, morpho-semantic, emotional expressivity or emotional sentiment polarity.

**Fig. 5:**
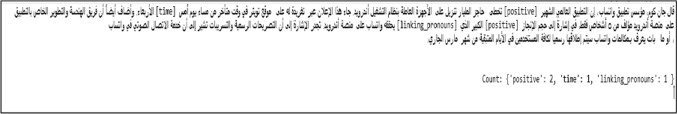
Example of tagged text file

Figure 5 shows an example of a text with the polarity and lexical words tagged, with the summary of the taggers below.

**Fig. 6:**
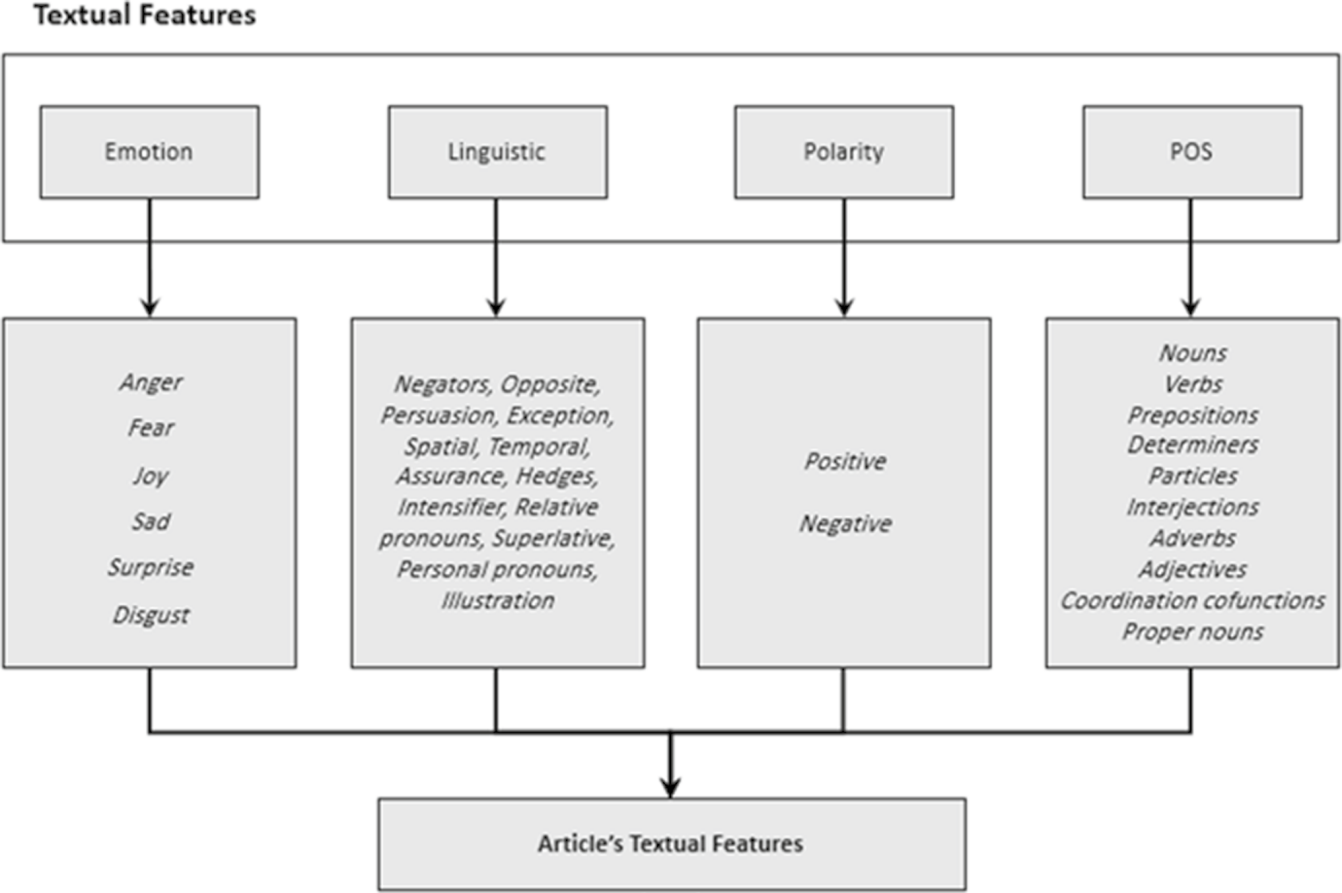
Set of textual features

In total, we extracted four textual features from all 549 real and 549 fake Arabic articles in the dataset (Fig. 6).

## Results and Discussion

Although we did not propose a new classification method in this study, we use the collected features to provide more accurate Arabic classifiers. In this section, we will discuss the lexical densities in the dataset for each textual feature. Then, we display our model’s prediction performance against each feature category individually and combined. Finally, we tested the prediction rate of our model against human performance.

### Dataset

Our dataset includes 1098 records with 549 records classified as ‘false’ and 549 records classified as ‘real’ articles. The dataset distribution is shown in Table 3 using lexical densities concept. Lexical densities are the total number of lexical words divided by the total number of words [59]. We calculated the average lexical density for each textual feature category with all the datasets in our work. We found that the number of verbs appeared more in fake news than in real news. This supports the findings of Rayson et al. [21], cited in Sect. 2.1.2.2 regarding imaginative text compared to informative text and one of the studies conducted by Zhou et al. [60] on deception detection, but not all of them [61].

**Table 3:** Lexical density of each feature category

Feature category	Feature	Fake articles (L%)	Real articles (L%)
Emotional expressiveness (E)	Anger	0.67	0.48
	Sad	0.268	0.25
	Fear	0.232	0.21
	Joy	1.08	1.40
	disgust	0.056	0.03
	Surprise	0.175	0.16
Totals		2.481	2.53
Syntactic–semantic roles (R)	Assurance	0.734	0.43
	Negations	0.49	0.35
	Illustration	0.06	0.04
	Relative Pronouns	0.794	0.85
	Intensifiers	0.21	0.12
	Hedges	0.533	0.39
	Causation	1.596	1.20
	Temporal	0.853	0.65
	Spatial	0.514	0.50
	Exclusive	0.133	0.14
	Personal pronouns	0.077	0.05
	Superlative	0.364	0.45
	Contradiction	0.49	0.08
Totals		6.848	5.25
POS (S)	Noun	41.40	44.16
	Verb	9.49	9.30
	Preposition	7.61	7.37
	Determiner	0.97	0.72
	Particle	0.45	0.34
	Interjection	0.01	0.00
	Adverb	0.90	0.68
	Adjective	10.70	11.12
	Coordinating conjunction	6.37	4.60
	Proper noun	9.64	10.59
Totals		87.75	88.88
Contextual polarity (P)	Negative	0.20	0.16
	Positive	0.36	0.31
Totals		0.56	0.47
Total features	31		

### Analysis of Lexical Density of Each Textual Category

Overall, as previously demonstrated, fake articles contained lower emotional words compared to real ones. So, it can be said that the participants made an effort to avoid over-wording the fake articles with emotional words in an attempt to make them appear real. Furthermore, additional analysis has revealed that negative emotions, including sadness, fear, and anger, increased in the fake articles. The negative polarity found therein supports this insight: it is clear that the negative words increased in that category compared to the real articles. Having said this, positive words also increased in fake articles. So, this may lend credence to Li et al.’s [62] idea that deceivers often tone down their articles in order to conceal their real aims. This is achieved via increasing positive/negative words in existing positive/negative articles. What is more, we found that causations, negations, assurance, intensifiers, hedges, and contradictions were frequently used in fake articles. After all, the participants aimed to make their articles ‘genuine’ by using the aforementioned linguistic functional terms, specifically by incorporating these terms as naturally as possible, all while avoiding exaggeration, which could potentially expose the deceit. On the one hand, in relation to POS, nouns and adjectives were frequently used in real articles compared to fake ones. This could result from the articles being about Hajj. This is a religious performance that, importantly, has a wide range of adjectives and nouns. On the other hand, associated adverbs and verbs were much more frequently used throughout the fake articles compared to the real ones. This finding supports Newman et al.’s [63] belief that liars, as opposed to those speaking the truth, often use more verbs in order to provide concrete yet simple descriptions of their false statements.

### Training of Deception Detection

Our research applied NB, RF, and SVM classifiers. The selection of these machine learning algorithms is due to their performance identifying fake news in similar models (NB [26], RF [64], SVM [65]), and are considered adequate in many classification applications. NB classification technique is based on Bayes’ Theorem with an assumption of independence among deception features, which holds for our 31 features. RF is an ensemble learning method for classification method based on training a multitude of decision trees and providing the predictor based on the classes’ mode or mean prediction of the individual trees. SVM is a set of algorithms that analyses data for classification and regression analysis and is commonly used in machine binary machine learning. All methods were compared to a baseline (random), where an article is assigned a class randomly (Table 4).

**Table 4:** Classifier performance

Classifier	Feature	AUC	F1	Precision	Recall
Random (baseline)	–	0.50	0.50	0.50	0.50
Naïve Bayes (NB)	E	64.7	61.9	62.2	62.0
	E + R	71.2	65.4	65.9	65.5
	E + P	65.7	60.1	61.0	60.5
	E + R + P	71.6	65.9	66.4	66.1
	E + S	69.5	64.4	64.4	64.4
	E + R + S	72.2	67.0	67.2	67.1
	E + P + S	69.7	64.9	64.9	64.9
	R	68.4	62.9	63.4	63.1
	R + P	68.8	62.6	62.9	62.7
	R + S	72.0	67.6	67.7	67.6
	R + P + S	72.2	67.3	67.4	67.4
	P	55.2	54.5	54.5	54.5
	P + S	67.7	64.0	64.0	64.0
	S	67.3	64.2	64.2	64.2
Random forest (RF)	E	61.3	58.9	59.7	59.3
	E + R	74.1	67.8	67.8	67.8
	E + P	66.3	61.9	61.9	61.9
	E + R + P	75.5	68.7	68.7	68.7
	E + S	72.0	65.1	65.3	65.2
	E + R + S	84.3	78.2	78.2	78.2
	E + P + S	74.8	68.2	68.4	68.3
	R	73.5	68.4	68.5	68.4
	R + P	74.0	68.9	69.0	68.9
	R + S	85.0	78.6	78.6	78.6
	R + P + S	85.3	**79.0**	79.0	79.0
	P	52.6	54.6	54.8	54.8
	P + S	66.5	62.3	62.8	62.6
	S	62.9	58.7	59.1	58.9
Support vector machine	E	49.6	47.9	51.4	51.0
	E + R	48.1	58.7	58.8	58.7
	E + P	50.0	50.8	52.0	51.8
	E + R + P	50.8	58.1	58.2	58.1
	E + S	57.4	56.0	56.9	56.5
	E + R + S	67.7	62.7	63.3	62.9
	E + P + S	59.6	56.9	57.4	57.2
	R	41.3	57.8	58.2	58.0
	R + P	49.4	58.8	59.1	58.9
	R + S	66.7	60.9	61.4	61.1
	R + P + S	67.2	61.7	62.5	62.0
	P	51.4	47.4	47.4	47.4
	P + S	57.0	56.1	56.3	56.2
	S	53.2	55.3	55.8	55.6

E = Emotional expressivity, R = syntactic**–**semantic roles, P = contextual polarity S = part of speech

We used the Scikit-learn library in our experiment. To validate our results, we applied 70% training 30% testing validation. For feature selection, we used the information gain method. Since the data size was small, we ran our experiment on a standard computer with 1 TB and 7 io dual core processor. We report Area Under Curve (AUC), Precision, Recall, and F1 measures.

The contribution of the syntactic**–**semantic roles seems to have been the most productive for our model. F1 measure was 68.4%, 73.5%, 41.3% for NB, RF, SVM, respectively. The performance of RF was higher by approximately 15% than NB and by 22% from SVM. RF built balanced random trees from the 31 linguistic features, where all of them were equally distributed between the dataset, except for causation 1.6%. Similar results could be deduced for Emotional Expressiveness where F1 measure was 61.9%, 58.9%, 47.9% for NB, RF, SVM, respectively; however, since the densities were not high, NB was performing slightly better than the RF algorithm. P did not provide any valuable contribution compared to the baseline, which was 54.5%, 54.6%, 47.4% for NB, RF, SVM.

Therefore, combining S and R increased the accuracy of the classifiers 67.6%, 78.6%, 60.9%, which are NB, RF, SVM. RF was able to build ensemble decision trees based on two different categories of features yielding the highest performance with an increase of approximately 10.2% compared to S alone and by 16.7% compared to R alone. The best performance was achieved by combining 25 features of R, P, and S. We note that the combined features of E, R, and P resulted in 67.0%, 68.7%, and 62.7% for NB, RF, and SVM. Also, combining all the investigated 31 features yielded F1 measure for RF accuracy of 78%.

The dominant features for deception detection were the linguistic features, which yields the best performance (79%) compared to all other features. This is compatible with the Pérez-Rosas, et al. study [7] where the psycholinguistics feature category was the most dominant in detecting fake news.

Our findings show that the model’s best prediction accuracy involved combining the textual features: R, P, and S. Though 79% marks a high level of accuracy, there is room for improvement. We also found that our finding is consistent with Pennebaker and King [23] that only words, conjunctions, and prepositions are associated with cognitive complexity, and the few bias markers, hedges, and subjective terms have been found useful [66]. Also, the results are consistent with peculiar POS lexical markers [67]. Our findings support the study that deceivers portrayed a more significant expressivity based on nouns and adjectives consistent with Zhou [68]. Overall combining significant deceptive features enhances the classifiers.

### External Validation Using Human Performance

To measure the relative value of using an automated deception detection classifier for news articles, we compared it to unaided human judgment. For this test, we decided to focus on satirical news articles. One reason for this was that satirical news relies on distorting legitimate news making a one-to-one balance of fake news to real news straightforward for collecting the dataset.

An objection may be made that satire is different in kind than more nefarious fake news and that deceptive intent and the accompanying psychological states are not present. While this is true, the reason so much study has been devoted to training automated detection classifiers to differentiate between satirical news and fake news is because the two are so similar [31]. Therefore, we expect our classifier to perform similarly in this test. Moreover, satire most often relies on the reader’s knowledge of the satirical nature of the source. In social media, where articles are forwarded without the context of the website itself, accurate human judgment is required for it not to be harmful. Presumably, satirical news would be the one subset of fake news where human performance should be the best, thus raising the stakes for the accuracy of our classifier.

**Fig. 7:**
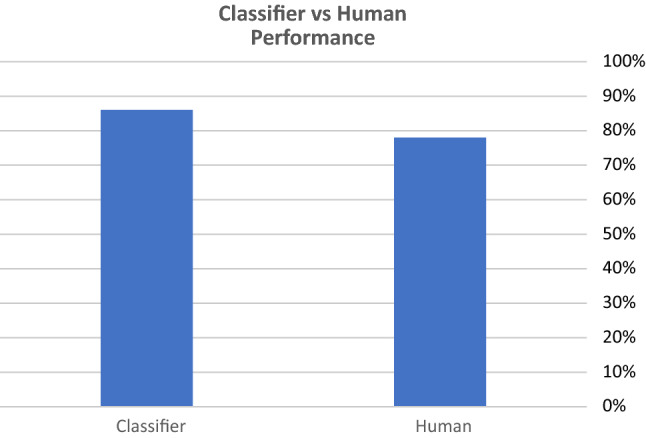
Classifier versus human performance

First, we collected fifty news articles from Arabic fake satire news website targeted at political issues.^6^ Then, we matched each false article with legitimate article from popular Arabic news agencies.^7^ In total, we had fifty fake articles, and corresponding to them were fifty real articles. We tried as much as possible to balance our dataset by matching each fake article with a real article that has the same length and details the same event or character in the fake article. The articles were distributed manually to 28 participants from our local university, with our work background. We asked them to label each article as real or fake based on their judgment. The same dataset was uploaded to the model, and the performance of our model was evaluated compared to human performance. Since we had narrowed down the best performance of our model to the RF model with 31 features, this was the model used in the test. The results showed that our RF model outperformed humans. Our model classified 86% of the articles correctly, while 23 humans classified 78% correctly and the rest of 6 classified less than that correctly. This highlights the fact that humans continue to struggle with deception detection and that automated computational linguistic aids like this can prove valuable in aiding humans in this task (Fig. 7).

## Conclusion and Discussion

In this work, we have addressed the detection of Arabic fake news based on textual analysis. Our proposed model achieved more than 75% accuracy in predicting the veracity of Arabic news articles. To achieve this, we firstly collected real news articles from reliable news sources. Secondly, to imitate the fake news production in the real world, real news articles were manipulated into fake articles using crowdsourcing. Third, four textual features categories were extracted using an Arabic natural language processing tool designed by the researchers. Arabic linguistic wordlists were organized and professionally reviewed for use in our work and for future Arabic textual analysis projects. The textual features extracted were emotion, linguistics, polarity, and part of speech. Finally, these features were used to train our model to detect deceptive text, in our case fake news. As a result, we evaluated the model’s prediction accuracy which gave promising results, 78%. Our most intriguing finding is that linguistics features extracted were the most dominant cues used to detect deceptive text in fake news. Our model can be considered a significant step forward in detecting Arabic fake news. This is something that should be borne in mind in future studies with more textual features, when investigating new approaches to detect Arabic fake news.

## Footnotes

1 http://www.mideastmedia.org/survey/2017/chapter/social-media/#s225.

2 http://norumors.net/.

3 Falsoo.com.

4 https://akeed.jo/ar/.

5 https://fatabyyano.net/.

6 www.alhudud.net.

7 www.okaz.net, www.sabq.net, www.youm7.net.
